# Investigating the Effect of Rock Bridge on the Stability of Locked Section Slopes by the Direct Shear Test and Acoustic Emission Technique

**DOI:** 10.3390/s20030638

**Published:** 2020-01-23

**Authors:** Qifeng Guo, Jiliang Pan, Meifeng Cai, Ying Zhang

**Affiliations:** School of Civil and Resource Engineering, University of Science and Technology Beijing, Beijing 100083, China; guoqifeng@ustb.edu.cn (Q.G.); caimeifeng@ustb.edu.cn (M.C.); b20160024@xs.ustb.edu.cn (Y.Z.)

**Keywords:** rock bridge, rock slope, energy, acoustic emission, slope stability

## Abstract

As a portion of intact rock separating joint surfaces, rock bridge plays a significant role in the stability of rock slopes. This paper aims to investigate the effect of different rock bridges on the mechanical properties and failure mode of rock slope by means of the direct shear test and acoustic emission technique. Field conditions were simulated in direct shear tests which were carried out on specimens with rock bridges at different continuity rates, normal stress, arrangements, and joint angles. Experimental results indicate that the strength of specimens is controlled by the rock bridge and the structural plane. The rock bridge contributes to the strength of the specimen, while the through plane weakens the strength of the specimen. The increase of normal stress can weaken the stress concentration near the tip of the rock bridge and improve the shear resistance of the specimen. The different arrangement of rock bridge has little effect on the normal displacement of the specimen, and has a great influence on the shear strength. The shear capacity of the specimen is related to the angle of the crack, and the angle of the crack is approximately proportional to the peak shear strength. For the specimens with different joint occurrence, the mode of crack propagation at the initial stage is basically the same, and the specimen is finally damaged due to the generation of through cracks in the core area of rock bridge. The instantaneous release of the huge energy generated during the experiment along the shear direction is the root cause of the sudden failure of the rock bridge. The formation, aggregation, and transfixion process of rock bridge is of concern and has been experimentally investigated in this paper for the prevention and control of the locked section rock slope with sudden disasters.

## 1. Introduction

A large number of high and steep rock slopes consist of intact rock and joints. A rock bridge is a portion of intact rock separating joint surfaces, which can also be called a locked section. The joints which are not fully connected and persistent for the existence of rock bridges are called non-persistent joints. The non-persistent joints widely exist in slope, chamber and other rock masses, which plays an important role in the stability of rock mass [1,2]. The rock bridge or locked section plays a key bearing role on the potential sliding surface of the slope and bears any stress concentration. The slope whose stability is controlled by the locked section is called the locked section slope [2]. At present, in the field of mining and geotechnical engineering, the locked section rock slopes are widely distributed and numerous, and their failure evolution process presents strong gradualness and abruptness. Therefore, it is of great significance to carry out a stability analysis of such slopes [3,4].

The strength and deformation of the locked section determine the overall stability of the slope. The stress concentration caused by slope excavation leads to the continuous expansion and penetration of joints to the position of rock bridge. The penetration failure of rock bridge is usually the main cause of rock slope instability [5,6]. The cracks propagation, coalescence, and penetrating through in the rock bridge will eventually lead to the failure of rock mass, which can be termed as “rock bridge failure”. Further, “rock bridge failure strength” is proposed as the peak-strength for the rock bridge cracking through [7,8]. Chen et al. [8] conducted conventional triaxial tests on rock specimens with different rock bridge lengths to study the acoustic emission (AE) characteristics during the process of rock bridge through failure under different stress paths, as well as the influence of confining pressure and the rock bridge length on the AE characteristics. Han et al. [9] studied the crack growth and rock bridge through mode under different rock bridge lengths, and the inclination through the biaxial cyclic load test. Xi et al. [10,11,12] investigated the crack initiation, coalescence, and propagation processes for concrete materials. Then, they quantitatively analyzed the functional relationship between the through strength, the rock bridge dip angle, the friction coefficient of the structural plane, the confining pressure, the connectivity rate and the structural plane dip angle, and proposed the rock bridge through criteria with two non through structural planes. However, most of the above research focuses on the strength criterion and the classification of the expansion mode of the locked section rock mass. There is a lack of systematic and in-depth study on the mechanism of the sudden failure during the progressive failure process of the locked section rock mass and the influence of the occurrence of the locked section on the failure mode of the rock mass.

The initiation and propagation of stress induced crack damage is a necessary precursor to all brittle failure of rocks [13]. Therefore, indirect monitoring methods, such as the AE and microseismic (MS) monitoring techniques, have been introduced in rock engineering for rock damage assessment both in the laboratory and in situ. In both AE and MS monitoring, the objective is to monitor brittle deformation remotely by recording the sounds emitted when rock cracks or fractures slip. AE and MS techniques have been used by many, e.g., in mining for roof fall prediction [14], and in civil engineering for rockburst risk assessment [15,16], rock slope stability [17], and large-scale underground cavern stability monitoring [13]. The MS monitoring is suitable for wide-range monitoring (can cover hundreds of m^3^) and detecting induced large-scale cracks, which can be employed for large rock mass stability evaluation, whereas the AE monitoring is suitable for analyzing local failure within a small volume (less than one cubic meter) of rock mass and investigating the evolution process of large-scale fracture from small-scale cracks generation and coalescence [18]. The monitoring of AE in the laboratory has proven to be a powerful tool available to study the brittle failure of rocks in a controlled stress environment.

AE is a phenomenon involving the development of transient elastic waves caused by rapid energy release due to crack growth [19]. Kaiser was the first to indicate an effect of rock stress history on the production of acoustic emissions. AE can be monitored when the load exceeds the maximum history load experienced by the rock. But there is an absence of AE for loads smaller than the previous maximum stress [20]. Goodman confirmed that rock also has a Kaiser effect through experiments, which laid a foundation for the application of AE technology in rock mechanics [21]. Early AE technique has been applied in security problems of metal mines, coal mines, and tunnel engineering. The research of rock AE technology mainly focuses on two aspects. On the one hand, combined with the experiment, the rock fracture mechanism is studied by analyzing the AE signal. On the other hand, AE technology is used to monitor and predict the stability of engineering rock mass. Rocky landslides contain a lot of rock bridges, and research on rock bridge were carried out by experimental methods and simulated by analytical and numerical simulation techniques. Wang et al. [22] carried out an experimental study on the AE source location in square cylinder granite specimens under uniaxial compression. Yang et al. [23] used triaxial compression test to test the strength, deformability, failure behavior, and AE positions of red sandstone. Moradian et al. [24] evaluated the AE signals generated during direct shear test on different types of joints. Chen et al. [25] carried out small-scale direct shear tests of rock bridge with different lengths and a large-scale landslide model with locked section, and analyzed the relationship between AE events and record time during the tests.

In this paper, specimens with different joints are first made using rock-like materials according to the principle of similarity physical simulation. Direct shear tests are carried out on the specimens to investigate the effects of the continuity rate, normal stress, arrangements, and angle of the rock bridge on rock failure. The expansion and penetration process of the specimens are observed and monitored by using a digital camera and AE detection technology. The research reveals the influence of the different occurrence of rock bridge on the mechanical properties and failure mode of the locked section rock mass, and explores the mechanism of the high-speed sliding of the locked section slopes.

## 2. Experimental Approach

### 2.1. Specimen Preparation

As a kind of homogeneous quasi-brittle material, concrete can be used to make rock-like specimens [12,26]. In order to make jointed rock-like specimens, firstly insert steel piece into the concrete specimen, and take out the steel piece after the initial setting of the concrete specimen. Place the specimen at room temperature for 24 h, and then put it into a constant temperature and humidity curing box for 7 days. In order to better observe the generation, expansion, and penetration of cracks in the specimen during loading, the prefabricated cracks are filled with gypsum and stored 24 h before a direct shear test. The length, width, and height of the specimens need to be 100 mm, 100 mm, and 100 mm, respectively. Taking sand and rubber powder as aggregates, the real rock is simulated by controlling the amount of rubber powder and water cement ratio. Rubber powder is used to replace sand aggregate with 0%, 5%, 10%, and 15%, and the water cement ratio is 0.3, 0.4, and 0.45, respectively. In order to improve the adhesion between rubber powder and cement mortar, the rubber powder needs to be treated with 1% NaOH solution.

**Figure 1:**
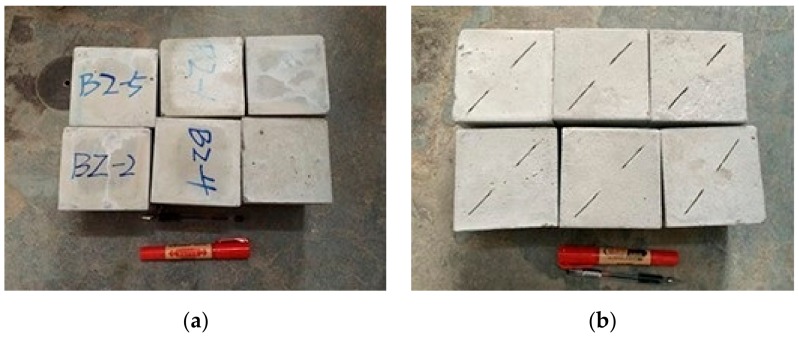
Some rock-like specimens. (**a**) intact rock-like specimens, (**b**) jointed rock-like specimens.

The density of these specimens was measured, and the conventional uniaxial test and splitting tensile test were carried out. During the experiment, the whole process of specimen failure was observed and compared with that of real rock specimen. Finally, the water cement ratio of 0.4 and rubber powder content of 10% were selected. Based on the physical and mechanical experiments of five rock groups in the Washan stope, when comparing the mechanical indexes of the specimens under each ratio, it is found that the ratio scheme determined in this paper can better meet the experimental requirements of similar materials. According to the proportioning scheme of similar simulated materials determined in this paper, the specimens are cured for 7 days after pouring, and then the basic mechanical properties of rock-like materials are determined. Some rock-like specimens are shown in Figure 1, and the test results of mechanical properties are shown in Table 1.

### 2.2. Testing Schemes

Rock mass is composed of relatively complete rock blocks, joints, and fracture networks. Many scholars have conducted a lot of uniaxial and biaxial compression experiments on non-persistent joints specimens. Such experiments can be used to study the initial extended through stage of specimens, but they cannot study the stage after the peak, so the experiments have great limitations and incompleteness [27,28,29]. The direct shear test can make up for the deficiency of uniaxial and biaxial compression loading tests. After the external load exceeds the initial crack strength, it can continue to load, and obtain the shear strength curve, maximum shear strength, and residual strength after the peak of the shear test [30,31]. The modified triaxial shear rheological testing machine is used for the test, which is mainly composed of the host system, servo control system, and hydraulic system. The direct shear test is completed by means of automatic loading normal stress and tangential stress. By directly installing an AE sensor to monitor the energy release during the whole process of specimen failure, the mechanism of sudden failure of locked section rock mass is revealed from the perspective of stress and energy. This test is mainly carried out according to four schemes. Under the condition that other conditions remain unchanged, the influence of rock bridge on the locking effect of the locked section specimen is studied under a different continuity rate, normal stress, arrangement mode of rock bridge, and joint angle of the locked section. In order to reduce the test error, six specimens of each type were made.

**Figure 2:**
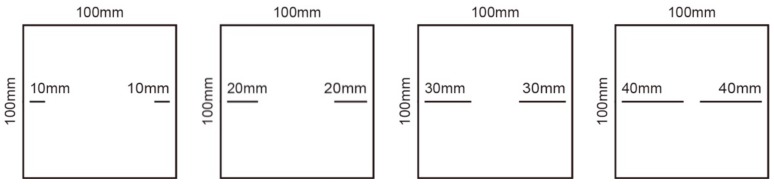
Schematic diagram of joint specimens in scheme I.

Scheme I: The effect of different continuity rate on the shear strength of specimens is studied by inserting steel plates. The specimens with joint length of 10 mm, 20 mm, 30 mm, and 40 mm were made, respectively, and the corresponding continuity rates were 0.2, 0.4, 0.6, and 0.8, respectively. This is shown in Figure 2.

**Figure 3:**
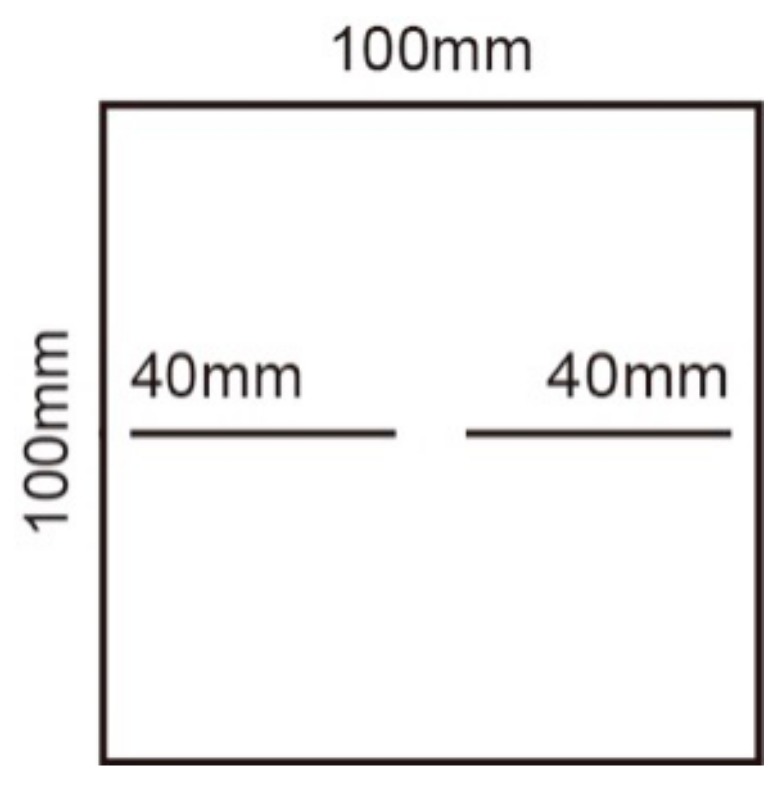
Schematic diagram of joint specimens in scheme II.

Scheme II: The collinear specimen with a continuity rate of 0.8 is made by inserting steel pieces. The direct shear tests with normal stress of 0.5 MPa, 1 MPa, 2 MPa, and 3 MPa are respectively carried out on the specimen. The influence of different normal stress on the failure of the specimen is studied. The specimen is shown in Figure 3.

**Figure 4:**
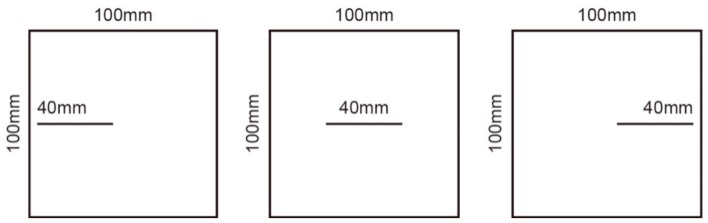
Schematic diagram of joint specimens in scheme III.

Scheme III: Adopt the method of inserting steel pieces, respectively make specimens with a continuity rate of 0.4, lock section on the left, joint in the middle and lock section on the right, and study the influence of different arrangement of joints and lock section on the failure and penetration of specimens, as shown in Figure 4.

**Figure 5:**
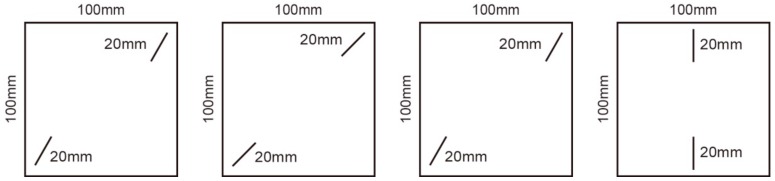
Schematic diagram of joint specimens in scheme IV.

Scheme IV: On the basis of other conditions remaining unchanged, the specimens with joint dip angles of 30°, 45°, 60°, and 90° are made to study the influence of different joint dip angles on the failure and penetration of the specimens, as shown in Figure 5.

### 2.3. Testing Procedures

**Figure 6:**
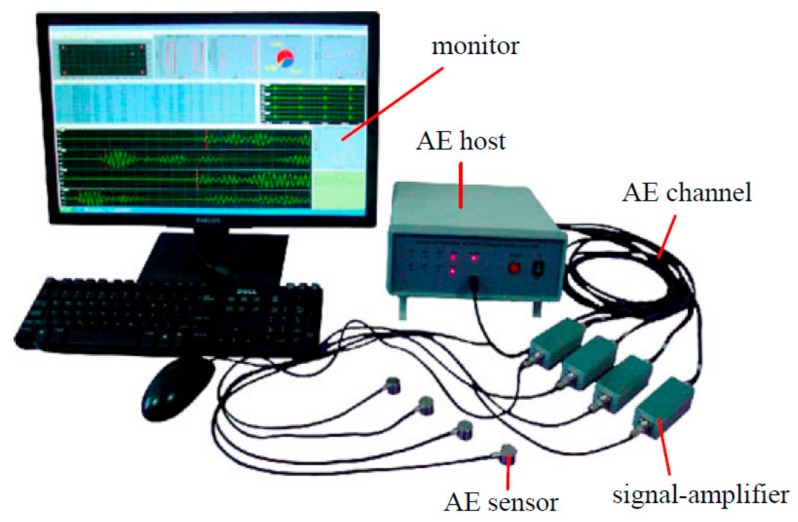
AE signal analyzer (DS2-8A).

**Figure 7:**
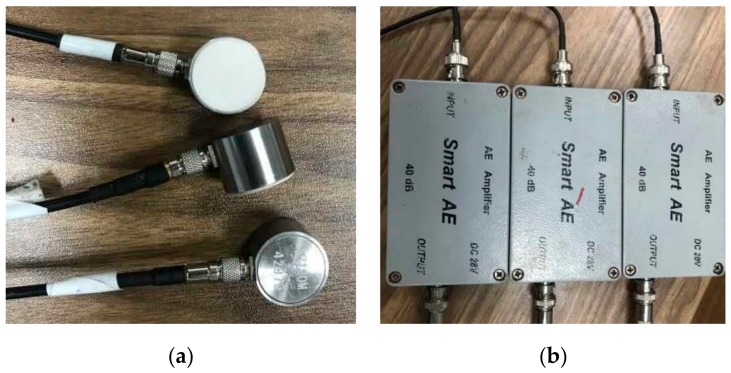
AE sensors and AE amplifiers. (**a**) AE sensors, (**b**) AE amplifiers.

The experimental equipment is a RLJW-2000 microcomputer controlled triaxial shear rheological testing machine. During the experiment, an AE signal analyzer (DS2-8A) is used to detect and locate the energy release and failure process of specimens during the shearing process. The experimental instrument is shown in Figure 6. The diameter and height of the AE sensor used in this test are 18.8 mm and 15 mm, respectively. The detection surface contacted with the specimen is ceramic material. The sampling frequency range of the AE sensor is 50–400 kHz, and the applicable temperature range is between −20 °C and −130 °C. The preamplifier is a 40 dB gain adjustable amplifier, which can amplify the signal 100 times. The AE sensors and amplifiers are shown in Figure 7a,b.

**Figure 8:**
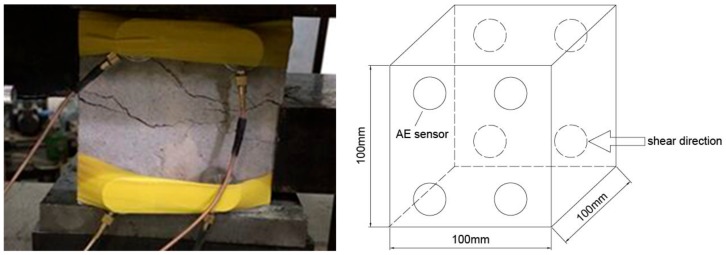
Location of the acoustic emission (AE) sensors.

The specific operation steps of the whole test process are as follows:

(1). Test preparation: turn on the direct shear test instrument for preheating for about 5 min, turn on the computer for system inspection, and start the hydraulic device when all indicators are normal.
(2). AE instrument debugging: connect the cable, amplifier and AE sensors of the AE instrument, turn on the AE instrument and the computer, set the AE signal acquisition threshold voltage to 100 mV, and the sampling frequency to 3 MHz, and use the lead-off method to confirm whether the AE instrument is normal. After debugging, turn off the AE instrument.
(3). Place the specimen: place the specimen in the fixture as required to ensure that the upper and lower clamps are aligned. After the specimen is placed, the loading plate is laid on the upper part of the specimen.
(4). Install the displacement sensor: install the displacement sensor in the vertical direction and the horizontal direction according to the requirements, and adjust the displacement sensor to the appropriate position according to the instructions of the main control computer. In order to ensure the accuracy of displacement, two displacement sensors are installed in vertical direction and horizontal direction, respectively, and the displacement accuracy of the sensor is 0.001.
(5). Installation of AE sensor: apply appropriate an amount of Vaseline between the specimen and AE sensor, and stick the AE sensor onto the surface of the specimen. The specific location of AE sensors is shown in Figure 8. In order to prevent the sensor from falling off during the test, eight AE sensors are pasted at the front and back of the specimen, and all the sensors are fixed with adhesive tape.
(6). Operation of direct shear test: in order to make the indenter and specimen in good contact, it is necessary to preload the specimen. Through the load control normal loading process, at the beginning of the laboratory test, the load is added to 5 kN according to the preloading rate of 0.5 mm/min, and then the load is added to the predetermined value according to the loading rate of 5 kN/min. The loading process in the tangential direction is controlled by deformation. The load is preloaded to 5 kN in the tangential direction according to the preloading rate of 0.5 kN/min, and then the load is stopped when the tangential displacement reaches 5 mm according to the loading rate of 0.3 mm/min. In order to keep the data synchronization between the direct shear test system and the AE test system, both systems need to start recording at the same time.
(7). End of test: after the specimen is damaged, stop loading and close the AE instrument before the data of the direct shear instrument and the AE signal file are saved. Remove the AE sensor, then remove the specimen from the direct shear apparatus, observe the damage of the specimen and take photos for preservation. Prepare for the next specimen.

## 3. Experimental Results Analysis

According to the four experimental schemes, the direct shear tests were carried out on the locked section specimens with different continuity rate, normal stress, arrangement of rock bridges and joint angles. During the experiment, the normal stress curve and tangential stress curve of the specimens under each scheme are recorded. According to the experimental results, the locking mechanism and joint weakening mechanism of the rock bridge under the condition of micro experiment are studied, and the application of the experimental results in the macro engineering is analyzed.

### 3.1. Effect of Rock Bridge Locking on Strength

**Figure 9:**
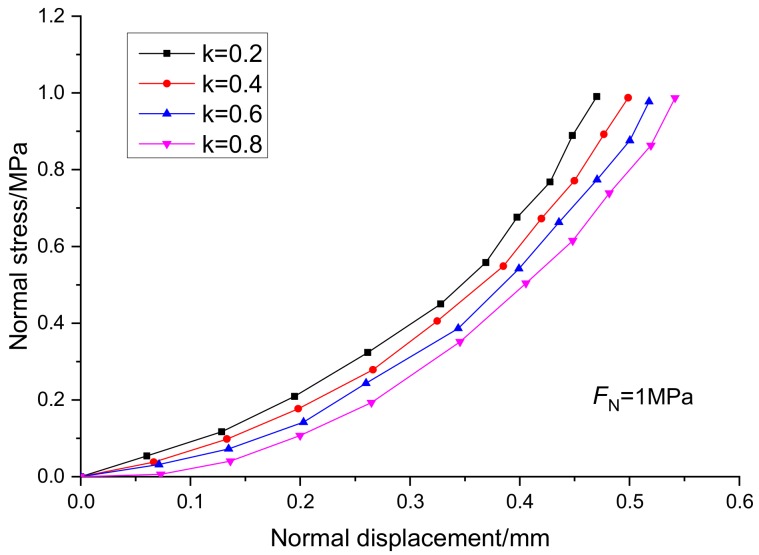
Relationship curves between normal displacement and normal stress at different continuity rates.

**Figure 10:**
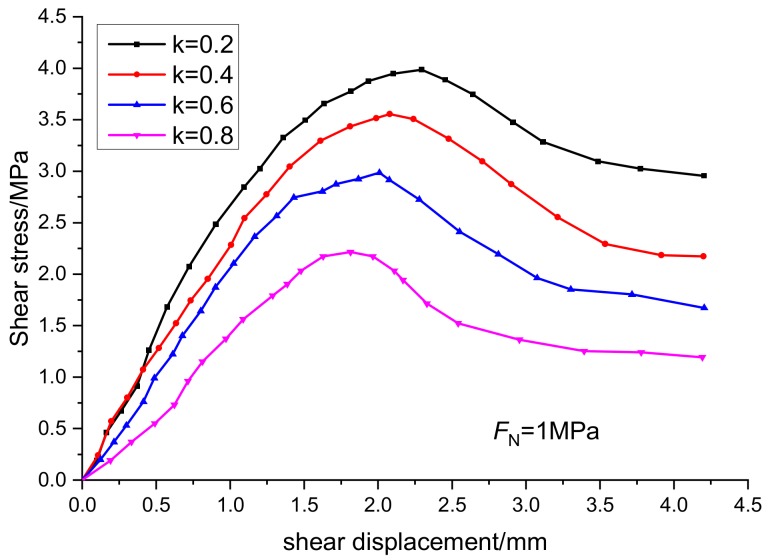
Relationship curves between shear stress and shear displacement at different continuity rates.

Under the condition of normal stress of 1 MPa, the relationship curves between stress and displacement of specimens with joint continuity rates of 0.2, 0.4, 0.6, and 0.8 are shown in Figure 9 and Figure 10. The joint continuity rate is expressed by k.

It can be seen from Figure 9 and Figure 10 that the normal displacement of the specimen increases with the increase of the normal stress, and presents a hyperbolic growth mode. Under the same normal stress condition, the greater the continuity rate is, the greater the normal displacement of the specimen is. Under the same shear stress condition, the greater the continuity rate is, the greater the shear displacement of the specimen is, while the influence of the continuity rate on the tangential stress curve is greater than that of the normal stress curve. According to the experimental results, the strength of rock mass is controlled by the rock bridge and structural plane. Therefore, when studying the strength of rock mass in practical engineering, it is necessary to fully consider the weakening effect of discontinuities such as faults and joints on the strength of rock mass.

**Figure 11:**
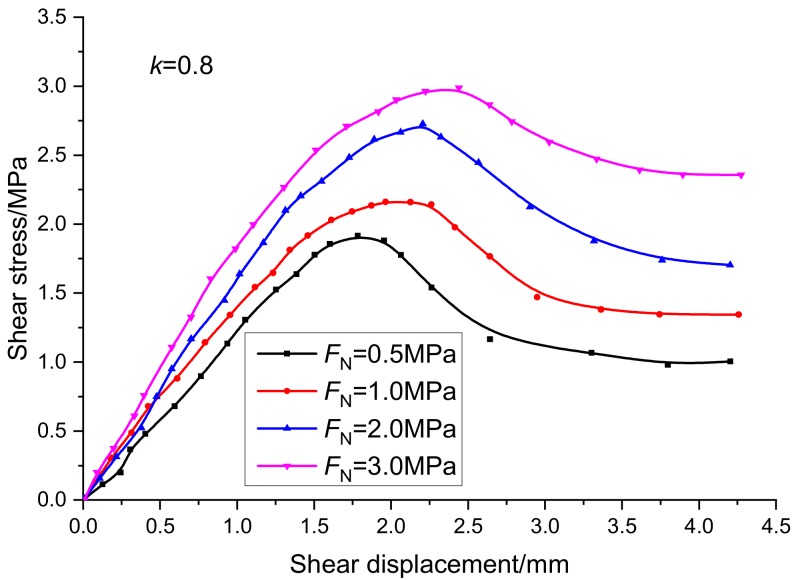
Relationship curves between shear stress and shear displacement under different normal stress conditions.

The tangential deformation curve of the specimen with continuity rate of 0.8 under normal stress of 0.5 MPa, 1 MPa, 2 MPa, and 3 MPa is shown in Figure 11. The normal stress is expressed by *F*_N_. When the normal stress is 0.5 MPa, the peak tangential displacement of the specimen is 1.7 mm and the peak shear strength is 1.8 MPa; when the normal stress is 3.0 MPa, the peak tangential displacement of the specimen is 2.6 mm and the peak shear strength is 3.1 MPa. With the increase of normal stress, the friction force on the crack surface increases gradually, which can weaken the stress concentration near the rock bridge tip and improve the shear resistance of the specimen.

**Figure 12:**
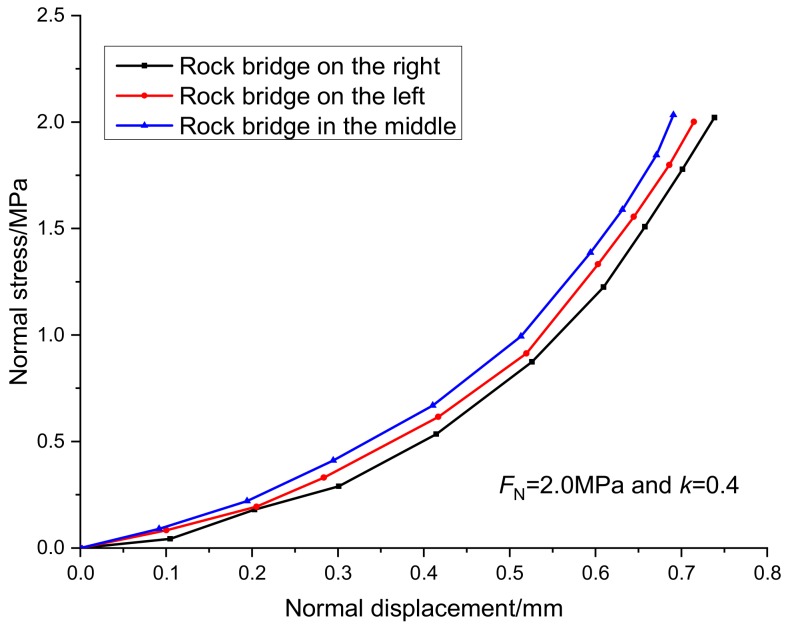
Relationship curves between normal stress and normal displacement under different arrangement of the rock bridge.

**Figure 13:**
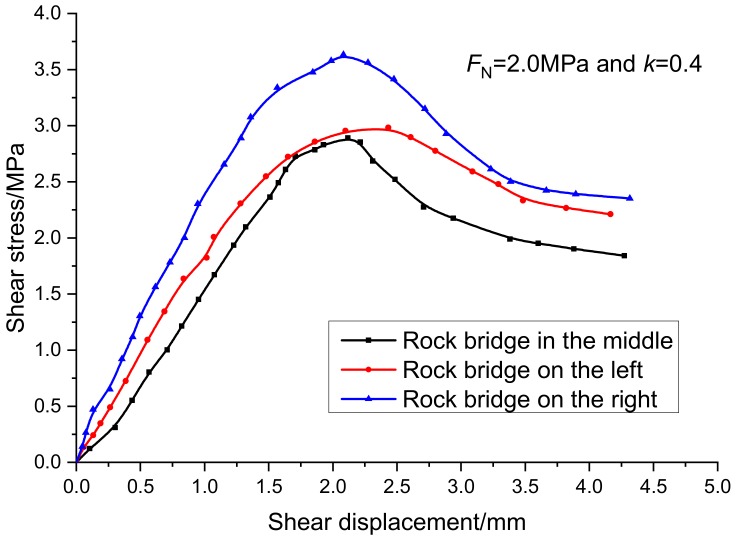
Relationship curves between tangential stress and displacement under different arrangement of the rock bridge.

When the continuity rate is 0.4 and the normal stress is 2 MPa, the normal deformation curve and tangent deformation curve of the specimens with different rock bridge arrangements are shown in Figure 12 and Figure 13. Under the same experimental conditions, the equivalent crack length of the specimen in the horizontal direction is the same, the normal displacement of the specimen with the crack in the middle is the smallest, and the normal displacement of the specimen with the crack on both sides is almost the same. However, the arrangement of joints and rock bridges has great influence on the peak shear displacement and peak shear strength. The peak shear displacement of rock bridge is 2.1 mm and the shear strength is 3.6 MPa. The peak shear displacement of rock bridge in the middle is 2.1 mm, and the peak shear strength is 2.9 MPa. The peak shear displacement and the peak shear strength of the rock bridge are 2.4 mm and 2.7 MPa, respectively. Therefore, the shear strength of rock bridge is the strongest when it is loaded at the far end. In the process of applying and transferring the shear force from left to right in a direct shear test, the friction on the fracture surface can weaken the stress concentration of the shear force at the rock bridge tip, enhance the shear resistance of the specimen, and make the specimen less prone to damage. In the actual engineering, the locking position of the structural plane in the whole rock engineering can be judged by observing whether the joints or faults are exposed and the penetration degree of the joints or faults, and the stability of the whole specific engineering can be distinguished and judged. Especially in the slope engineering, we should pay attention to the protection and reinforcement of the slope foot support, improve the shear resistance of the lock section, and maintain the stability of the slope.

**Figure 14:**
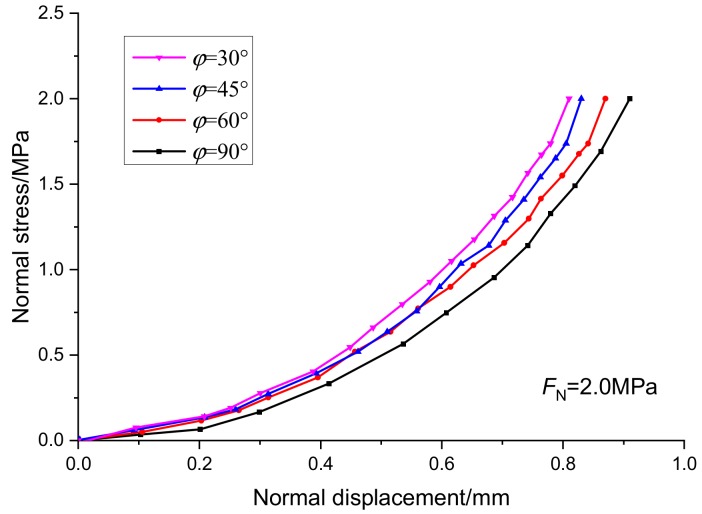
Relationship curves between normal stress and normal displacement under different joint dip angles.

**Figure 15:**
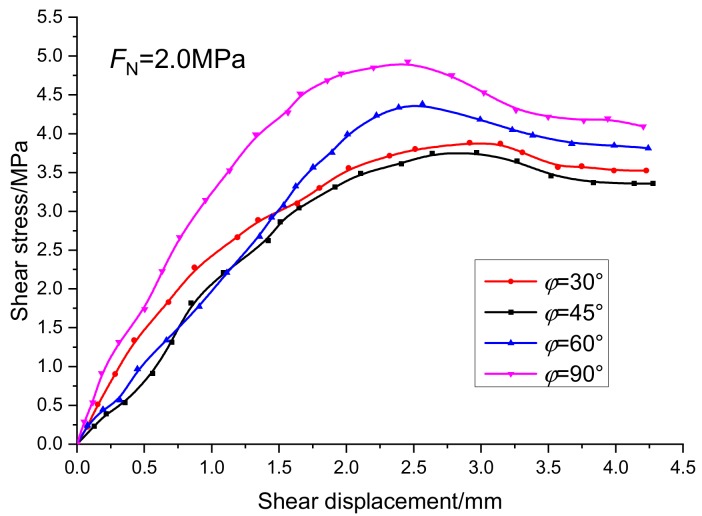
Relationship curves between tangential stress and tangential displacement under different joint dip angles.

According to scheme IV, the fracture rule and fracture characteristics of joints with different joint dip angle under compression and shear are studied. The joint dip angle is expressed by *φ*. The normal deformation curve and tangent deformation curve of the specimens are shown in Figure 14 and Figure 15. Under the same normal stress condition, with the increase of the fracture angle, the equivalent distance of fracture in horizontal direction increases gradually. Compared with the tangential displacement, the normal displacement is not greatly affected by the inclination of the fracture.

Figure 15 shows the relationship between the peak shear strength and the peak tangential displacement under different joint plane dip angles. When the fracture dip angle is 45°, the fracture dip angle is the same as the shear failure angle of the complete rock mass, so the peak shear strength of the specimen is the lowest, and the specimen is the easiest to crack. When the fracture angle of the specimen is 90°, the peak strength of the specimen is 4.9 MPa, the peak displacement is 2.46 mm, the weakening degree of the prefabricated fracture to the specimen is the smallest, the shear resistance of the specimen is the strongest, and the specimen is the least prone to fracture; when the prefabricated fracture angle is 30° and 60°, the shear resistance of the specimen increases gradually with the increase of the fracture angle. Its engineering significance is that the distribution of joints and fissures in the rock mass can be judged by the distribution characteristics of structural planes exposed on the rock mass surface, enabling us to make a reasonable assessment of the bearing capacity and stability of the whole project.

### 3.2. Analysis of the Process of Joint Extension and Transfixion

In the shear test of a certain normal stress, the crack initiation, propagation, and penetration of joints are the unique and dominant failure modes of rock. In the actual engineering, these groups of structural planes are mostly developed which are parallel or have a certain angle with the main axis of the project. Therefore, it is very likely that the expansion and penetration of the structural plane will lead to the formation of local connecting belt, which will cause the instability of the main body of the project. In this paper, the failure process of the specimens under loading is introduced by taking the specimens with a continuity rate of 0.4 as an example. In the experimental process, the main failure mode of the specimen is determined by observing the characteristics of secondary cracks.

**Figure 16:**
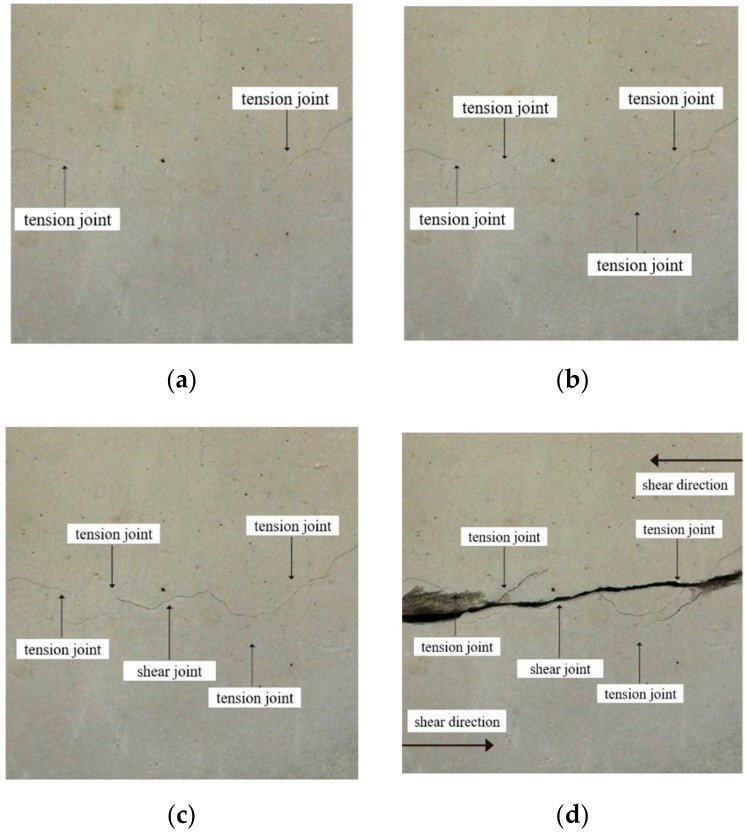
Shear failure of collinear cracks specimens. (**a**) Initial failure of the specimen, (**b**) Crack propagation failure, (**c**) Crack penetration failure, (**d**) Final failure of the specimen.

Figure 16a–c are the state diagrams of collinear non-persistent joints specimen in crack initiation stage, crack propagation stage, and crack through stage when the normal stress is 1 MPa. As shown in Figure 16a, at the initial stage of loading, with the increase of normal stress, the joint surface is gradually closed, and tension fractures appear at the outer tip of the fractures, and will develop in an arc-shaped direction. When the fractures develop to a certain extent, two cracks in an arc-shaped direction will also appear at the tip of the rock bridge, as shown in Figure 16b. As the shear stress continues to increase, the forward crack will close briefly under the action of normal stress, then the shear stress continues to increase, the crack gradually opens, and the forward crack expands. When the crack develops to a certain extent, a through crack will appear at the end of the crack, and the angle between the crack and the rock bridge is greater than 90°, as shown in Figure 16c. Before the specimen penetrates completely, the rock bridge area will form a “nuclear area” without fracture zone under the surrounding of the peripheral anterograde fracture and reverse fracture, and there will be no propagation crack in this area for a period of time, showing a blank state. When the shear stress continues to increase, there will be a through crack in the “core area” of the rock bridge, and the strength of the rock bridge will be completely lost, which shows a strong stage and sudden process. The generation, expansion and penetration of cracks in the core area of the rock bridge indicate the complete loss of the overall strength of the specimen, and the specimen mainly suffers from shear failure. The final failure mode of the specimen is shown in Figure 16d.

**Figure 17:**
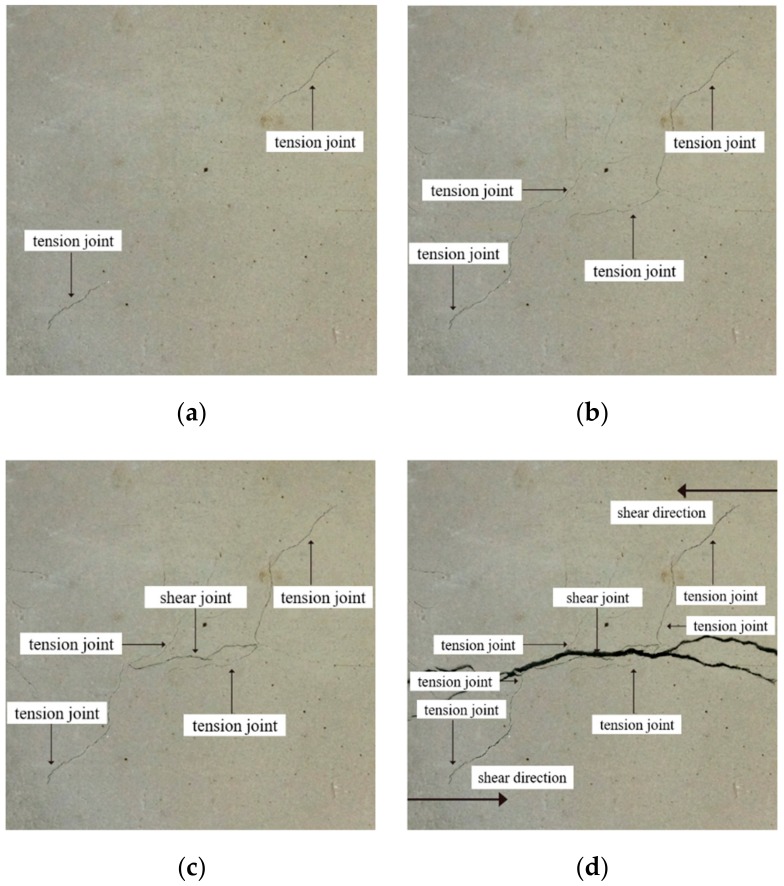
Shear failure of non-collinear cracks specimens. (**a**) Initial failure of the specimen, (**b**) Crack propagation failure, (**c**) Crack penetration failure, (**d**) Final failure of the specimen.

For the non-collinear cracks specimen, in the initial stage of loading, the outer end of the fracture will appear tension fracture, and continue to expand to the end of the specimen, as shown in Figure 17a. As the loading goes on, the end of the rock bridge will produce a longitudinal crack, as shown in Figure 17b. When the shear stress continues to increase, the crack growth rate slows down and will be closed from the open state for a short time. As the energy of the forward crack gradually releases, the reverse crack will be generated at the crack end, as shown in Figure 17c. When the shear force continues to increase, the cracks expand at the rock bridge, and gradually expand to the shear direction under the constraint of normal stress. The expanding crack and the new crack at the end of the crack converge in the rock bridge area, and then new cracks are generated. Finally, the failure occurs along the shear direction. The main failure of the specimen is the tensile shear composite failure, as shown in Figure 17d.

The joint mode of the collinear cracks specimen in the early stage of expansion is basically the same as that of the non-collinear cracks specimen. In the end, the failure of the collinear cracks specimen is also caused by the generation of through cracks in the nuclear area of the rock bridge. Under the condition of low normal stress, the collinear cracks mainly causes tensile failure, and under the condition of high normal stress, it mainly causes shear failure. The single failure mode of non-collinear cracks rock mass is less, and the main failure mode is tension shear composite failure. On the whole, the smaller the angle between the joint and the loading direction is, the later the new crack occurs, and the slower the propagation speed is, the greater the ultimate shear strength will be. For the specimens with the same joint attitude, the larger the normal stress is, the later the new cracks appear, the slower the specimen propagates and penetrates, the smaller the “core area” formed by the new cracks in the rock bridge is, and the greater the shear strength of the specimen becomes.

## 4. The Mechanism of Sudden Failure of Lock Specimen

The tensile failure of the micro units in the rock bridge is the main cause of the macro cracks. The continuous expansion and failure of the end of the rock bridge are consistent with the location of AE events. As shown in Figure 17, the breakthrough of the “nuclear area” fracture at the rock bridge marks the loss of the strength of the rock mass, and the energy released in this process can be characterized by AE counts. In order to study the relationship between the energy release and the difference between peak strength and residual strength during the instability of rock bridge, the difference between peak strength and residual strength during direct shear test is called the peak-residual strength drop, as shown in Equation (1). The ratio of the peak-residual strength drop to peak strength is called the peak-residual strength drop rate [32], as shown in Equation (2).

(1) $$\Delta \tau =\tau_{p}-\tau_{r}$$

(2) $$S_{d}=\frac{\Delta \tau }{\tau_{p}}=\frac{\tau_{p}-\tau_{r}}{\tau_{p}}$$

where, $\tau_{p}$ is peak strength, MPa. $\tau_{r}$ is residual strength, MPa. Δτ is the peak-residual strength drop, MPa. $S_{d}$ is the peak-residual strength drop rate.

The relationship between the peak-residual strength drop and AE counts under the loading conditions of scheme I and scheme II is shown in Table 2 and Table 3. 

**Figure 18:**
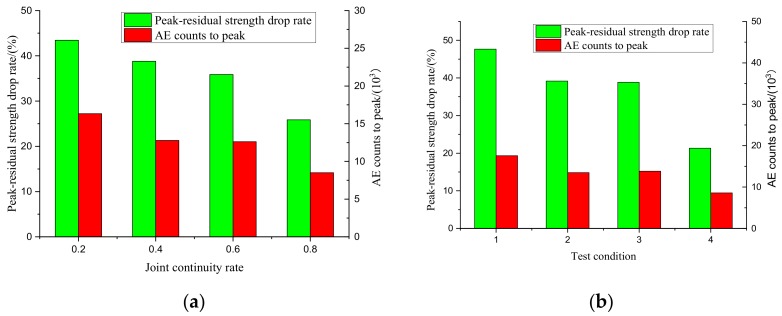
Relationship between the peak-residual strength drop rate and AE counts. (**a**) Scheme I, (**b**) Scheme II.

It can be seen from Figure 18 that when the core area at the rock bridge is formed, there will be a significant peak-residual strength drop in the rock mass, and the peak residual strength drop will produce a significant increase in AE counts. There is a consistent positive correlation between the peak-residual strength drop rate and AE counts. In the process of shearing, the residual strength of rock mass decreases rapidly by 21.33–47.64% compared with the peak shear strength, and the concentrated shear deformation of the specimen can be released instantaneously along the shear direction, resulting in the breakthrough failure of the specimen showing a strong burst.

**Figure 19:**
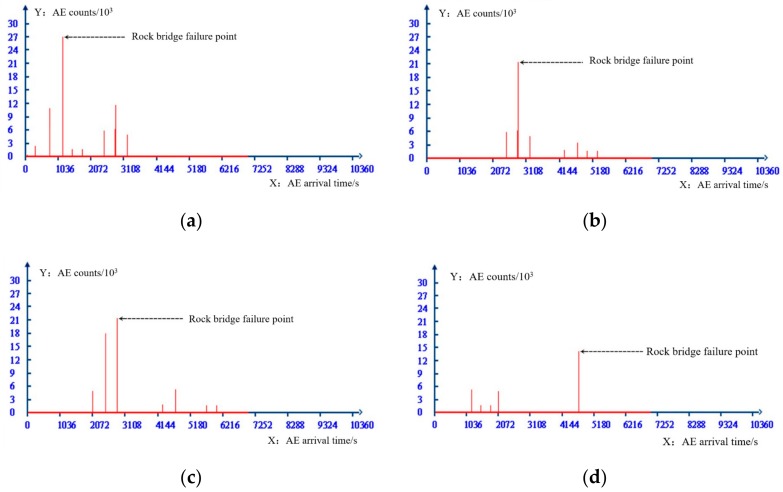
AE arrival time and AE counts under different joint continuity rates. (**a**) Joint continuity rate is 0.8, (**b**) Joint continuity rate is 0.6, (**c**) Joint continuity rate is 0.4, (**d**) Joint continuity rate is 0.2.

When the normal stress is 1 MPa, AE counts of specimens with different joint continuity rates are shown in Figure 19. It can be seen from the AE arrival time and counts that AE events is generated rapidly in a short time, and the peak strength of the specimen is transformed into the residual strength. It can be seen that the energy released by the peak-residual strength drop during the direct shear test is the internal cause of the sudden failure of the locked section specimen.

**Figure 20:**
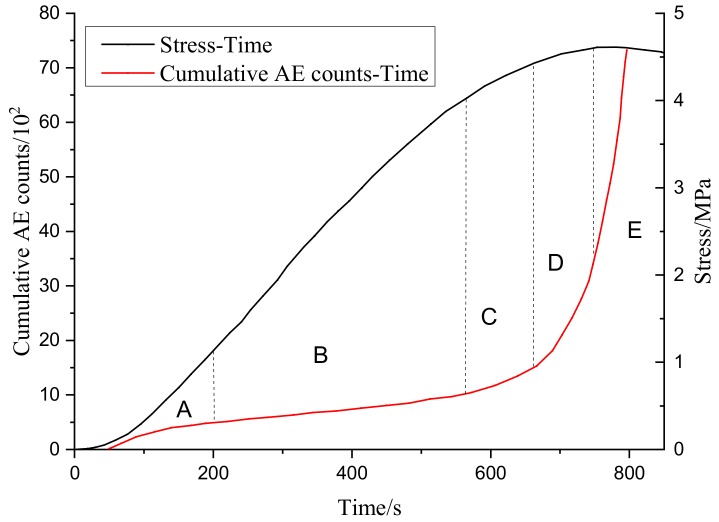
Change curve of tangential stress and change rule of cumulative AE counts.

**Figure 21:**
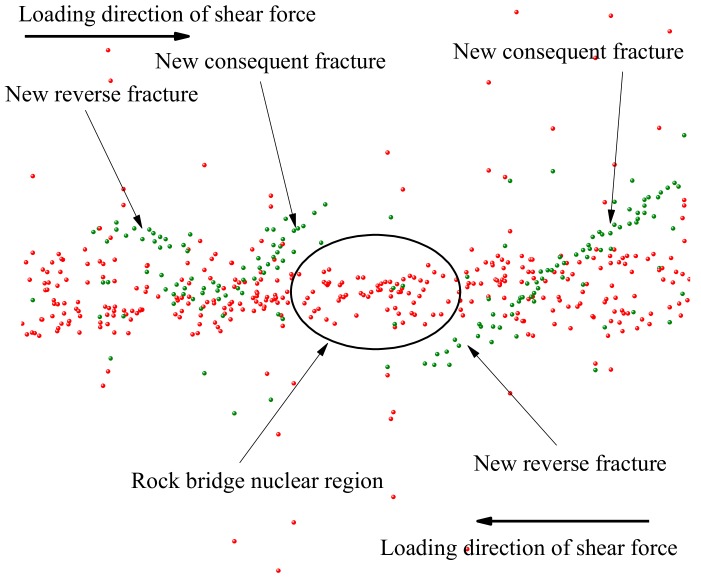
AE location of specimen during direct shear test.

The change curve of tangential stress and change rule of cumulative AE events of the specimen are shown in Figure 20. The AE positioning during the direct shear test of the specimen is shown in Figure 21. It can be seen that the failure and transfixion process of locked section rock mass has obvious stages, and the whole process has mainly gone through a fracture compaction stage (A), new fracture development stage (B), fracture instability and expansion stage (C), fracture transfixion stage (D), and specimen overall instability and sliding stage (E). In the fracture compaction stage (A), under the normal stress, the fracture surface is gradually compressed, and the silent AE signal is generated for a long time in this stage. With the increase of the shear force, only a small AE signal appears at the fracture end in the positioning area. This is because the fracture end is close to the loading end, so stress concentration will occur. In the development stage of the new fracture (B), with the increase of shear stress, the peripheral area of the rock bridge area successively appears forward fracture and reverse fracture according to the sequence. In this stage, the AE signal will first appear in the location area. With the increase of the loading shear stress, the new fracture continues to expand and run through, a large number of AE signals are generated in the blank area of the rock bridge, and the specimen enters the stage of fracture instability and expansion (C). In the stage of fracture instability (C), there will be a certain degree of rotation in the rock bridge area. During the rotation of the blank area of the rock bridge, the fracture surface immediately penetrated, and the specimen entered the fracture penetration stage (D). In the stage of fracture transfixion (D), there is a through fracture in the rock bridge area and a macro fracture surface in the rock mass, then the specimen enters the stage of overall instability and sliding (E). In the stage of global instability and sliding (E), with the increase of shear stress, the joint surface slides, and a large number of AE signals is generated in the rock bridge area. With the dislocation and sliding of the core like area of the rock bridge, the energy of the specimen is released rapidly, the AE signal increases linearly, and the strength of the specimen is lost. This stage mainly depends on the static friction force of the fracture surface to provide strength and stabilize at a certain value.

## 5. Conclusions

(1). The strength of the locked section specimen is controlled by the rock bridge and the structural plane. The rock bridge contributes to the strength of the specimen, while the transfixion plane weakens the strength of the specimen. The larger the continuity rate is, the more the strength of the specimen weakens. The increase of normal stress can increase the friction force of the joint surface, weaken the stress concentration near the rock bridge tip, and improve the shear resistance of the specimen. The different arrangement of the rock bridge has little influence on the normal displacement of the specimen, and has a great influence on the shear strength of the specimen.
(2). When the fracture angle is 45°, the peak shear strength of the specimen is the lowest and the specimen is most likely to fracture because the fracture angle is the same as the shear failure angle of the intact rock mass. When the angle of specimen crack is 90°, the weakening degree of prefabricated crack to specimen is the smallest, the shear resistance of specimen is the strongest, and it is the least easy to crack. When the angle of the prefabricated fracture is 30° and 60°, the shear capacity of the specimen increases with the increase of the angle of the fracture.
(3). The crack growth patterns of the specimens with different joint occurrences are basically the same at the initial stage, and at the end of the crack propagation, it is due to the generation of through cracks in the core area of the rock bridge. Under the condition of low normal stress, the tensile failure of the collinear cracks specimen mainly occurs, and under the condition of high normal stress, the shear failure mainly occurs. The single failure mode of non-collinear cracks specimens is reduced, and the main failure mode is tension shear composite failure. The sudden failure of locked section rock mass is caused by the sudden release of energy.
(4). The failure and transfixion process of the locked section specimen has obvious stages. The study on the formation, aggregation and transfixion process of the core area of the rock bridge has important guiding significance for the prevention and control of the locked section rock slope during sudden disasters.

## Figures and Tables

**Table 1:** Test results of the mechanical properties.

Bulk Density/(kN/m^3^)	Uniaxial Strength/MPa	Elastic Modulus/GPa	Poisson’s Ratio	Tensile Strength/MPa	Cohesion/MPa	Internal Friction Angle/°
19.7	26	2.7	0.2	2.6	3.90	42.1

**Table 2:** Relationship between the peak-residual strength drop rate and AE counts in scheme I.

Joint Continuity Rate	Peak Strength/MPa	Residual Strength/MPa	Peak-Residual Strength Drop Rate/%	AE Counts to Peak/10^3^
0.8	2.21	1.25	43.44	27.21
0.6	2.99	1.83	38.80	21.30
0.4	3.54	2.27	35.88	21.01
0.2	4.02	2.98	25.87	14.15

**Table 3:** Relationship between peak-residual strength drop rate and AE counts in scheme II.

Normal Stress/MPa	Peak Strength/MPa	Residual Strength/MPa	Peak-Residual Strength Drop Rate/%	AE Counts to Peak/10^3^
0.5	1.91	1.00	47.64	19.32
1	2.17	1.32	39.17	14.83
2	2.73	1.67	38.83	15.21
3	3.00	2.36	21.33	9.45
